# Robotic versus laparoscopic distal pancreatectomy: an up-to-date meta-analysis

**DOI:** 10.1186/s12893-017-0301-3

**Published:** 2017-11-09

**Authors:** Gian Piero Guerrini, Andrea Lauretta, Claudio Belluco, Matteo Olivieri, Marco Forlin, Stefania Basso, Bruno Breda, Giulio Bertola, Fabrizio Di Benedetto

**Affiliations:** 1 Department of Surgical Oncology. Surgical oncology Unit, National Cancer institute-Centro di Riferimento Oncologico IRCCS, Aviano (PN), Italy; 2 Hepato-Pancreato-Biliary Surgery and Liver Transplantation Unit, University of Modena and Reggio Emilia, Modena, Italy

**Keywords:** Pancreatic cancer, Distal pancreatectomy, Left pancreatectomy, Pancreatic resection, Robotic surgery, Laparoscopic surgery, Review, Meta-analysis

## Abstract

**Background:**

Laparoscopic distal pancreatectomy (LDP) reduces postoperative morbidity, hospital stay and recovery as compared with open distal pancreatectomy. Many authors believe that robotic surgery can overcome the difficulties and technical limits of LDP thanks to improved surgical manipulation and better visualization. Few studies in the literature have compared the two methods in terms of surgical and oncological outcome. The aim of this study was to compare the results of robotic (RDP) and laparoscopic distal pancreatectomy.

**Methods:**

A systematic review and meta-analysis was conducted of control studies published up to December 2016 comparing LDP and RDP. Two Reviewers independently assessed the eligibility and quality of the studies. The meta-analysis was conducted using either the fixed-effect or the random-effect model.

**Results:**

Ten studies describing 813 patients met the inclusion criteria. This meta-analysis shows that the RDP group had a significantly higher rate of spleen preservation [OR 2.89 (95% confidence interval 1.78-4.71, *p* < 0.0001], a lower rate of conversion to open OR 0.33 (95% CI 0.12-0.92), *p* = 0.003] and a shorter hospital stay [MD -0.74; (95% CI -1.34 -0.15), *p* = 0.01] but a higher cost than the LDP group, while other surgical outcomes did not differ between the two groups.

**Conclusion:**

This meta-analysis suggests that the RDP procedure is safe and comparable in terms of surgical results to LDP. However, even if the RDP has a higher cost compared to LDP, it increases the rate of spleen preservation, reduces the risk of conversion to open surgery and is associated to shorter length of hospital stay.

## Background

Distal pancreatectomy (DP) is the mainstay surgical procedure for the treatment of body-tail tumors of the pancreas [1]. This type of surgery, generally performed through an open access, a fairly common but potentially demanding procedure, is still burdened with a significant morbidity and mortality up of 5% [2, 3].

Laparoscopic distal pancreatectomy (LDP) is a relatively new procedure as compared with the well-established open distal pancreatectomy [4, 5]. The first LDP was in fact performed by Cuscheri in 1996 [6]. Since many authors still consider LDP to be a complex operation because of technical problems linked to the vascular control and dissection of the pancreatic gland that is deeply located in the retroperitoneum, this has resulted in a delay in the spread of LDP when compared to other mini-invasive surgical operations. Thanks to the improvement of technology and the experience gained in laparoscopic surgery, it has been shown that LDP has achieved oncological results comparable to open surgery, with an overlapping rate of morbidity, but with the advantage of small surgical incisions, shorter hospital stay and faster recovery [7–10].

The exceptional development of computer technology and its consequent biomedical applications have enabled the creation of robotic surgery. Robotic distal pancreatectomy (RDP) is the most recent frontier of minimally invasive surgery applied to the surgical treatment of pancreatic tumors [11, 12]. RDP was first performed by Melvin in 2003 [13].

Robotic surgery has theoretically made it possible to overcome the disadvantages of the laparoscopic approach to the pancreas. In fact, it allows optimal viewing through a three dimensional high definition surgical view, tremor filtration, large range of motion due to an internal articulated endo-wrist, all associated with remarkable ergonomics for the surgeon who performs the procedure [14].

Nevertheless, robotic procedures seem to be longer and have higher costs without a clear advantage in terms of surgical and oncologic outcomes [14, 15]. No randomized controlled trials (RCTs) comparing RDP and LDP have been published on this issue, only retrospective studies [16–26]. Moreover, none of these has reached a uniform conclusion in terms of efficacy and safety [27]. We therefore performed a systematic review and meta-analysis in order to compare the results of laparoscopic vs robotic distal pancreatectomy.

## Methods

### Literature search strategies and study selection

A systematic literature search was conducted independently by two authors (G.G. and A.L.) using the methods of the Cochrane collaboration. We searched the National library of Medicine (Medline, https://www.ncbi.nlm.nih.gov/pubmed/), the Cochrane central register of controlled trials (Cochrane library www.cochrane.org), and Embase (https://www.embase.com/login) for relevant articles published from January 1980 through December 2016.

The search strategy was set up using the key words or text words combined with a Mesh (Medical subject headings) database search. The terms used were: “Distal pancreatectomy”, “Pancreatectomy”, “Laparoscopic” and “robotic” with limitation of clinical studies and humans. The search was exploded using the related article term in Pubmed. In addition, the references of searched articles were manually cross-searched for additional publications, and captured citations were filtered for study design in order to identify all control studies. All data extraction was performed in duplicate. We included studies with more than five patients in each arm for comparison of clinical outcomes. Narrative reviews, case series or studies without matched groups were excluded. No unpublished data, or non-English manuscripts or data were used.

The methodological quality of case-control and cohort studies was assessed using the Newcastle-Ottawa scale [28]. Only studies that reached six points or more were considered qualitatively eligible for meta-analysis. To select the titles of relevant studies, abstracts and full text articles were screened.

### Quality assessment of the studies and inclusion criteria

We planned to include only randomized controlled trials (RCTs) in this review. However, we found no RCTs on the topic, so we performed a meta-analysis of observational studies. We included studies reported as full text, and studies published as abstract only. All studies with Robotic and Laparoscopic pancreatectomy were considered. Any etiology for distal pancreatectomy was eligible and there were no limitations because of race, gender or age. Two investigators independently reviewed the articles for eligibility and extracted data for the analysis. Any disagreement was resolved through discussion and consensus of the study team. The PRISMA criteria for reporting meta-analyses were used as guidelines in the construction of this analysis [29].

Distal pancreatectomy-related morbidities, such as pancreatic fistula (PF), bleeding rate and all Clavien-Dindo complications grade III or more were used as measures of outcome. Surgical results such as conversion rate, spleen preservation, operative time, length of hospital stay, oncological parameters, cost of operation were principal parameter analyzed in this the meta-analysis.

Data analysis was performed using the software Review Manager (RevMan) [Version 5.1. Copenhagen: The Nordic Cochrane Centre, The Cochrane Collaboration, 2011) and Metanalysis (Tecnopharma 2004 Italy). Results of the meta-analysis are presented as odds ratios (OR) with 95% confidence intervals (CI). The OR was used for dichotomous outcomes as the confirmatory effect size estimate. Continuous variables were analyzed using the weighted mean difference (WMD) with 95% CI. *P* values of < 0.05 were considered statistically significant. Heterogeneities of treatment effects between the trials were tested using *Q* statistics and total variation across studies was estimated by I2. A fixed-effect model was adopted if there was no statistically significant heterogeneity in this analysis. If the results of trials had heterogeneity *I* ^2^ > 50%, a random-effects model was applied, such as the DerSimonian-Laird method [30]. Potential publication bias was determined by conducting informal visual inspection of the funnel plot, but also using quantitative methods such as the test for asymmetry of the funnel plot.

### Definition of complications

Pancreatic fistula (PF) was defined as an amylase-rich fluid from the drain at biochemical evaluation or abnormal communication between the drain and the pancreatic anastomosis seen with fistulography. More recent studies used the definition of pancreatic fistula proposed by the International Study Group on Pancreatic Fistula (ISGPF), where the pancreatic fistula is defined as an output of any measurable volume of fluid with an amylase content greater than 3 times the upper normal serum value via an operatively (or subsequently) placed drain on or after postoperative day 3 [31]. Postoperative morbidity was defined as any complication in agreement with the Dindo classification [32]. Perioperative mortality was defined as death during the same hospital stay or within 90 days after discharge if the patient was discharged earlier.

## Results

### Included studies and patient characteristics

**Fig. 1:**
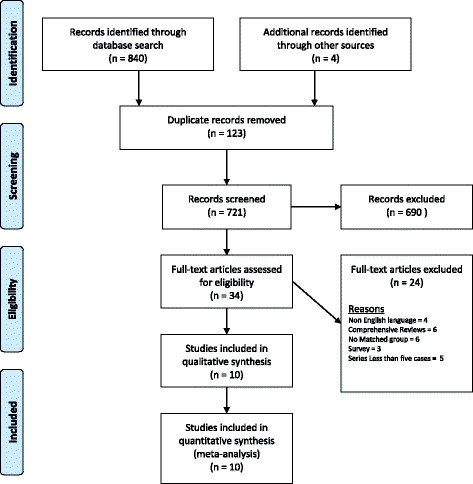
Prisma flow chart of the selection process

The initial search strategy retrieved 844 publications relevant to search words (Fig. 1). After screening all titles and abstracts, a total of 34 full papers were captured, of which 24 were excluded because of missing inclusion criteria. Ten comparative studies were identified for inclusion. The features and quality of the included studies are summarized in Table 1.

**Table 1:** Scale assessment of the quality of the studies

Author	Publication Year	Country	Study	Robotic distal pancreatectomy *n*	Laparoscopic distal pancreatectomy *n*	Newcastle – Ottawa scale Selection/comparability/ Exposure = total score
Balzano [16]	2014	Italy	Retrospective-Multicenter	31	140	4 / 1 / 2 = 7
Butturini [25]	2015	Italy	Prospective	22	21	4 / 2 / 2 = 8
Chen [17]	2015	China	Prospective	69	50	4 / 2 / 2 = 8
Daouadi [18]	2013	USA	Retrospective	30	94	4 / 2 / 2 = 8
Duran [19]	2014	Spain	Retrospective	16	18	4 / 2 / 2 = 8
Goh [20]	2015	Singapore	Retrospective	8	31	4 / 2 / 2 = 8
Kang [22]	2011	Korea	Retrospective	20	25	4 / 2 / 2 = 8
Lai [23]	2015	China	Retrospective	17	18	4 / 1 / 2 = 7
Lee [24]	2014	USA	Retrospective	37	131	4 / 2 / 2 = 8
Waters [26]	2010	USA	Retrospective	17	18	4 / 2 / 2 = 8

Ten studies describing 813 patients were identified for the meta-analysis Table 2. A total of 267 patients underwent robotic distal pancreatectomy and 546 patients laparoscopic distal pancreatectomy. The two groups were similar as regards demographics (age, body mass index (BMI), gender), comorbidities (American Society of Anesthesiologist score) and pathological characteristics. The number of patients in each study ranged from a minimum of 8 up to 140.

**Table 2:** Characteristic of included studies comparing robotic vs laparoscopic distal pancreatectomy, NA not reported

Author	Number of patients RDP vs LDP	Age RDP vs LDP	Female (%) RDP vs LDP	ASA (mean) RDP vs LDP	BMI RDP vs LDP	Malignant (%) RDP vs LDP
Balzano [16]	31 vs 140	Na	NA	NA	Na	18 vs 16
Butturini [25]	22 vs 21	54 vs 55	77 vs 71	1.91 vs 1.76	25.3 vs 24.1	13.6 vs 9.5
Chen [17]	69 vs 50	56.2 vs 56.5	67 vs 64	1.9 vs 1.94	24.6 vs 24.6	23.2 vs 22
Daouadi [18]	30 vs 94	59 vs 59	67 vs 65	2.9 vs 3.2	27.9 vs 29	43 vs 14
Duran [19]	16 vs 18	61 vs 58.3	44 vs 50	2 vs 1.9	Na	56 vs 44
Goh [20]	8 vs 31	57 vs 56	75 vs 62	1.2 vs 1	27.6 vs 23.9	0 vs 12.9
Kang [22]	20 vs 25	44.5 vs 56.5	60 vs 56	NA	24.1 vs 23.4	0 vs 48
Lai [23]	17 vs 18	61.2 vs 63.2	41 vs 78	NA	24.1 vs 25.7	64.7 vs 77.7
Lee [24]	37 vs 131	58 vs 58	73 vs 56	2.5 vs 3	28.7 vs 28.2	10.8 vs 14.5
Waters [26]	17 vs 18	64 vs 55	65 vs 50	2.9 vs 2.8	NA	29 vs 28

### Pancreatic fistula

**Fig. 2:**
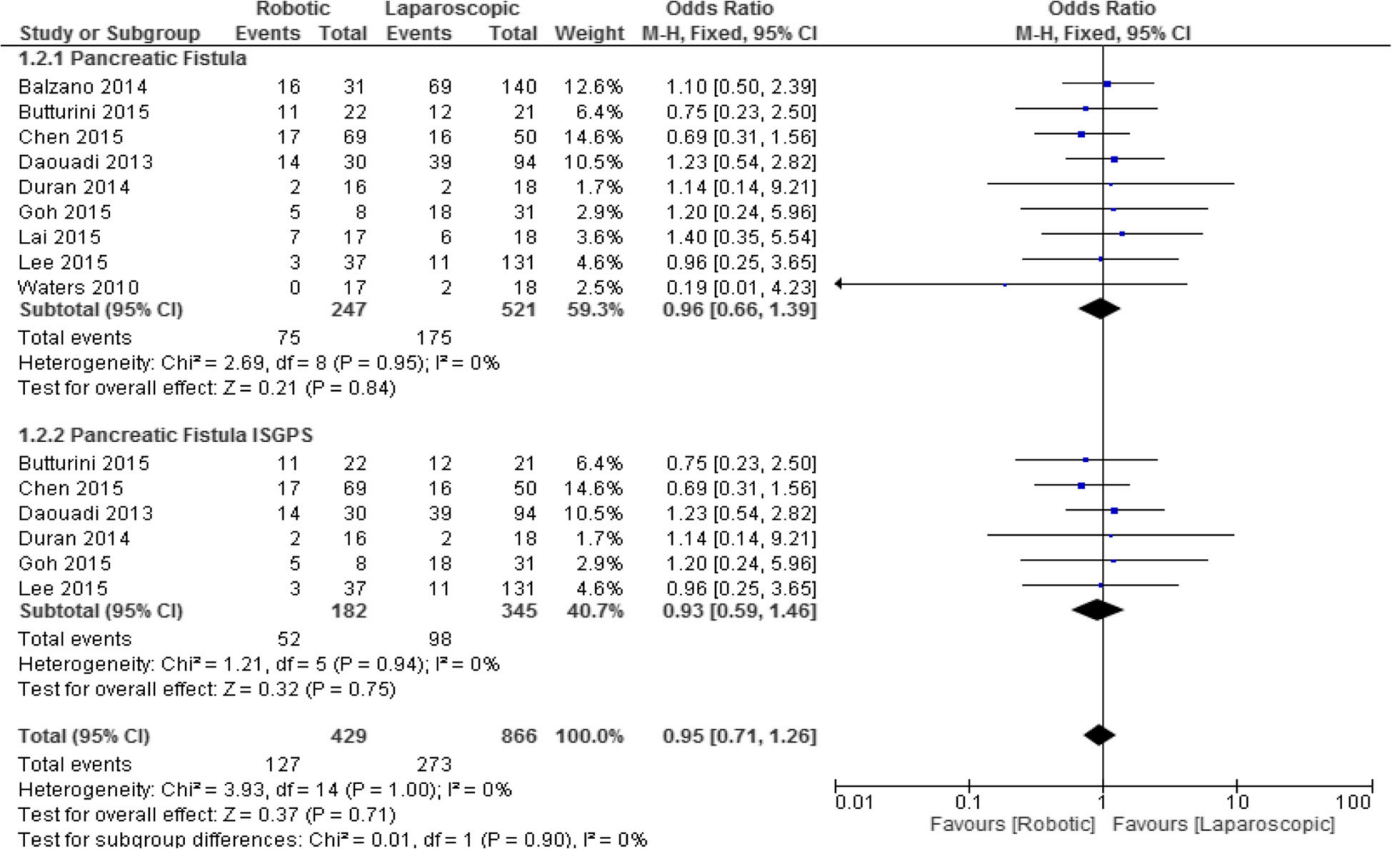
Forest plot displaying the results of the meta-analysis regarding pancreatic fistula

A fixed-effect model comparing the pancreatic fistula rate after RDP and LDP is shown in Fig. 2. At an Odds Ratio of 1 (central line) there is no difference in the rate of pancreatic fistula between the RDP and LDP groups. Values ​​greater than one represent an increased risk for pancreatic fistula in the Laparoscopic group, while a value less than 1 indicates a reduction in the risk of pancreatic fistula in favor of the Robotic group. A total of 768 patients were included in 9 articles. The rate of pancreatic fistula in the RDP group and LDP group was 30.3% (75/247) and 33.5% (175/521), respectively. The overall pooled results by meta-analysis revealed an OR 0.968 (95% confidence interval 0.66–1.39, *p* = 0.84). The funnel plot showed basic symmetry and the test for the asymmetry applied to the funnel plot was α = 0.24, *p* = 0.66 which suggested no publication bias. Specific subgroup analysis was performed in 6 studies that used a standard definition of pancreatic fistula according to the ISGPF classification. The rate of pancreatic fistula was 28.5% in (52/182) in RDP and 28.4% (98/345) in LDP. Meta-analysis showed no differences in the subgroups (OR 0.93 *p* = 0.75).

### Conversion rate

**Fig. 3:**
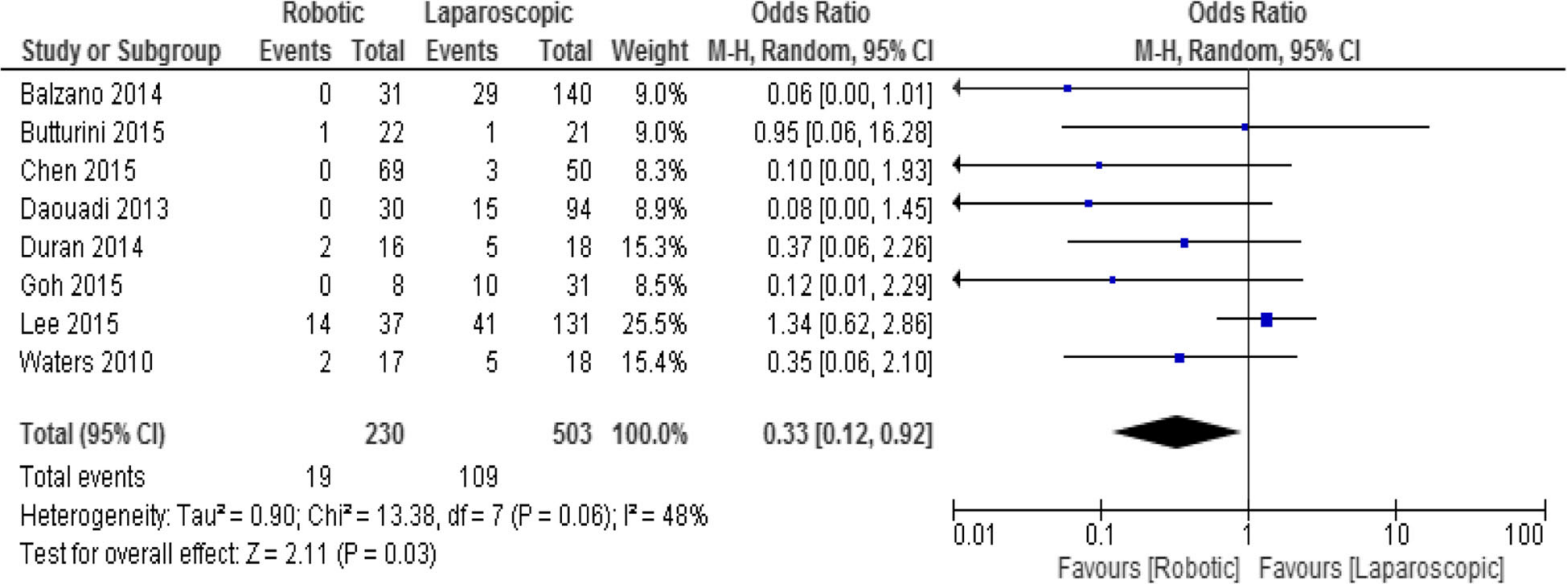
Forest plot displaying the results of the meta-analysis regarding conversion rate

Eight studies involving 733 patients reported the conversion rate. The conversion Rate in the RDP and LDP groups was 8.2% (19/230) and 21.6% (109/503), respectively. Meta-analysis showed a significant difference in the rate of the conversion between the two groups, with a lower rate in the RDP group (OR 0.33; 95% CI, 0.12–0.92, *p* = 0.03), Fig. 3.

### Spleen preservation rate

**Fig. 4:**
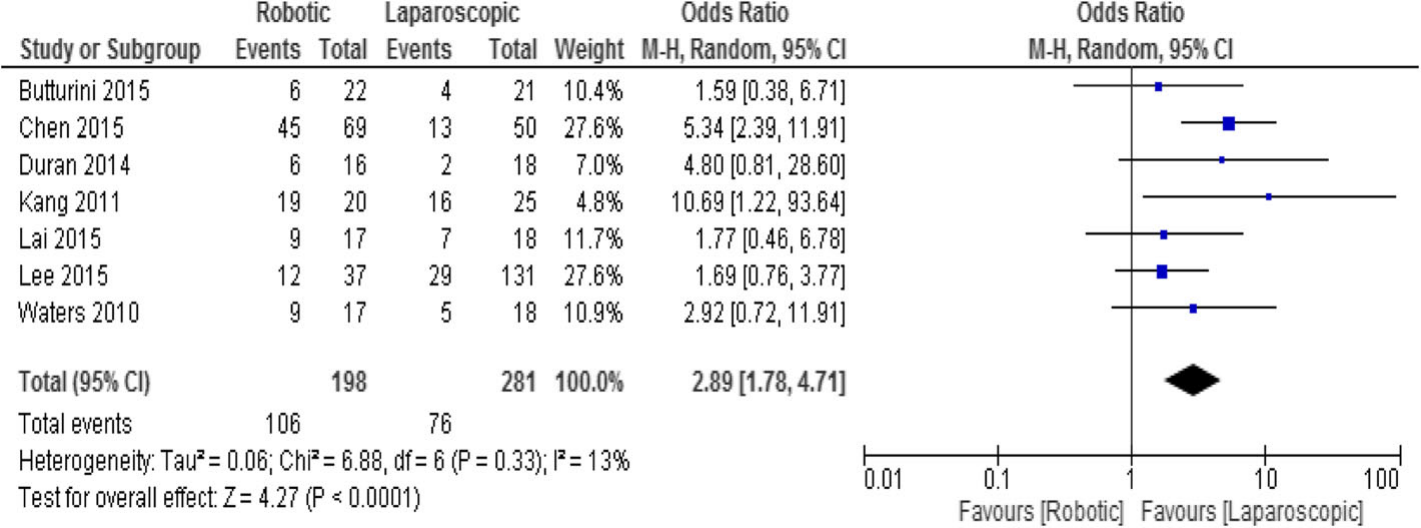
Forest plot displaying the results of the meta-analysis regarding spleen preservation rate

A total of 479 patients were included in 7 retrospective studies. Spleen conservation rate in the RDP and LDP groups was 48.9% (106/198) and 27% (76/281), respectively. Meta-analysis showed a significant difference in the rate of spleen preservation between the two groups with a higher rate in the RDP group (OR 2.89; 95% CI, 1.78–4.71, *p* < 0.0001), Fig. 4.

### Major morbidity (Clavien-Dindo classification ≥ III)

Nine studies involving 637 patients reported morbidity. The morbidity of the RDP and LDP groups was 16% (3/246) and 17% (67/391), respectively. Meta-analysis showed no significant difference in the rate of morbidity between the two groups (OR 1.19, 95% CI 0.73–1.91, *p* = 0.52), whereas 90-day mortality accounted for 1 death in both groups.

### Bleeding

The bleeding rate in the RDP and LDP groups was 8.2% (13/157) and 11% (23/5208), respectively. Meta-analysis of five trials showed no significant difference in the rate between the RDP and LDP groups (OR 0.8; 95% CI 0.41–1.79, *p* = 0.621).

### Oncologic parameters

Seven of nine studies reported the R0 margin status and six studies reported the number of harvested lymph nodes. All surgical specimens of RDP reported R0 negative margins, while 1% of LDP were diagnosed with positive margins. Five studies reported higher numbers of harvested lymph nodes in the RDP group, while two studies showed a higher number of lymph nodes in the LDP group (Table 3). Meta-analysis of the oncological variable could not be performed because of the impossibility to retrieve the standard deviation from the studies.

**Table 3:** Oncological characteristic of study population

Author	Lymph nodes Harvested Number LDP vs RDP	Rate of R1 resection Number of patients (% percentage) LDP vs RDP	Tumor size Centimeter (cm) LDP vs RDP
Balzano [16]	NA	NA	NA
Butturini [25]	15 (1–47 range) vs 11.5 (0–37)	0 vs 0	3.45 (1.5–1.7) vs 2.5 (0.5–9)
Chen [17]	9 vs 15	0 vs 0	3.5 (2.5–4 IQ) vs 3.5(2.1–3.5 IQ)
Daouadi [18]	9 (7–11 IQ) vs 19 (17–24 IQ)	5 (36) vs 0	3.4 ± 1.6 vs 3.1 ± 1.7
Duran [19]	5 ± 2 vs 12.5 ± 7.2	0 vs 0	4.1 ± 2.3 vs 2.9 ± 1.6
Goh [20]	NA	1(3.2) vs 1(12.5)	2.5 (0.8–7) vs 3 (1–6.9)
Kang [22]	NA	NA	3.0 ± 1.4 vs 3.5 ± 1.3
Lai [23]	NA	NA	NA
Lee [24]	10 ± 8 vs 12 ± 7	0 vs 0	NA
Waters [26]	11 vs 5	0 vs 0	3 ± 1 vs 2 ± 1

*NA* not reported

### Length of hospital stay

**Fig. 5:**
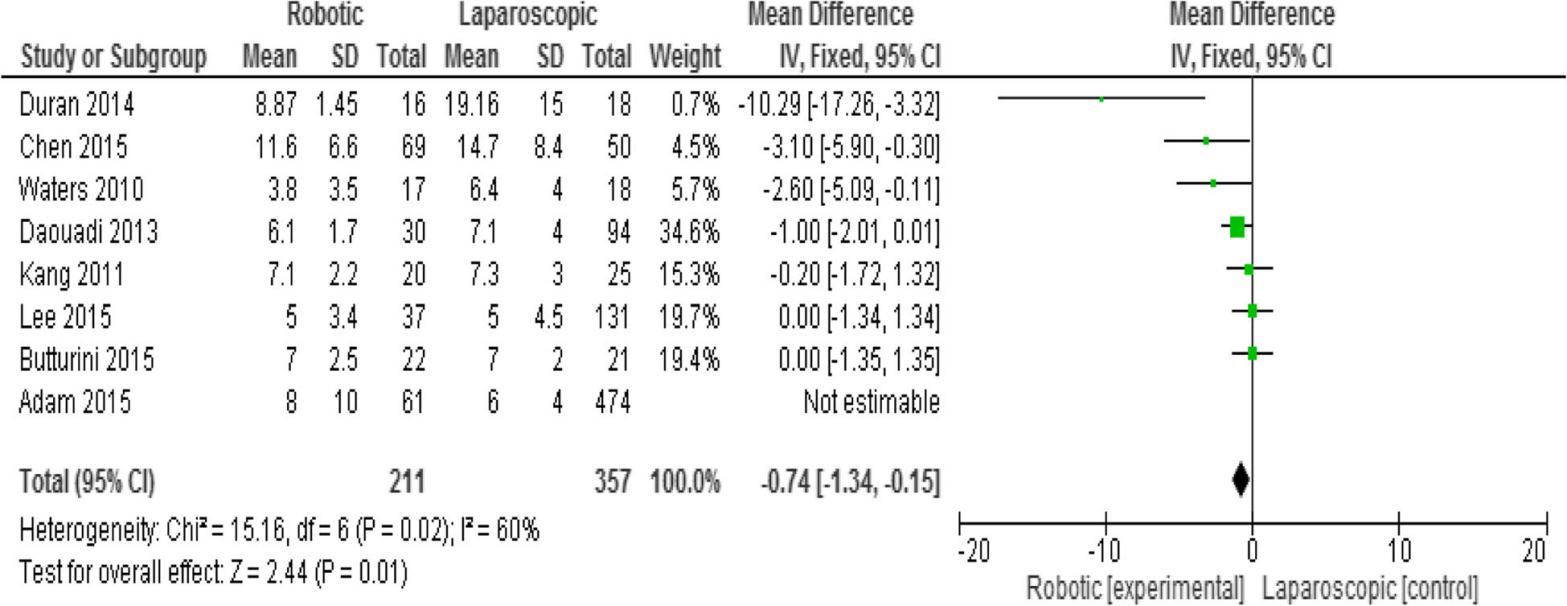
Forest plot displaying the results of the meta-analysis regarding hospital stay

Eight studies reported the length of hospital stay. The mean hospital stay was 7.18 days in the RDP group and 9.08 in the LDP group. Meta-analysis showed that the hospital stay was slightly shorter in the RDP group than in the LDP group with statistic significant difference (mean difference = -0.7495% CI -1.34 -0.15; *p* = 0.01), Fig. 5.

### Cost of the operation

**Fig. 6:**
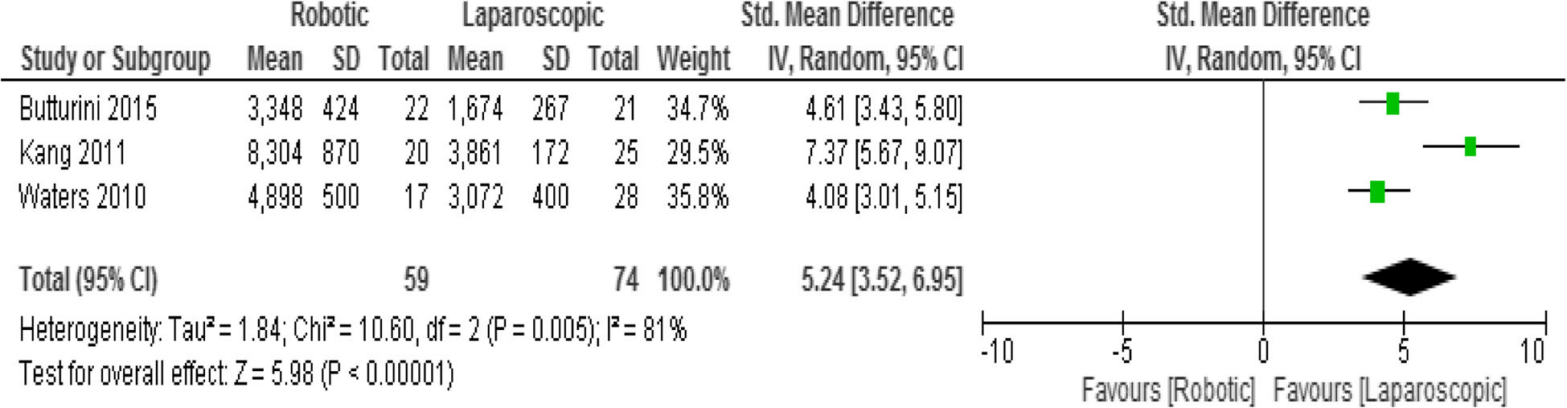
Forest plot displaying the results of the meta-analysis regarding cost of operation

Three studies reported cost analysis. Meta-analysis showed that the cost of the operation was higher in RDP group (Standard mean difference 5.24, 95% CI 3.52 -6.95, *p* < 0.00001), Fig. 6.

### Operative time

**Fig. 7:**
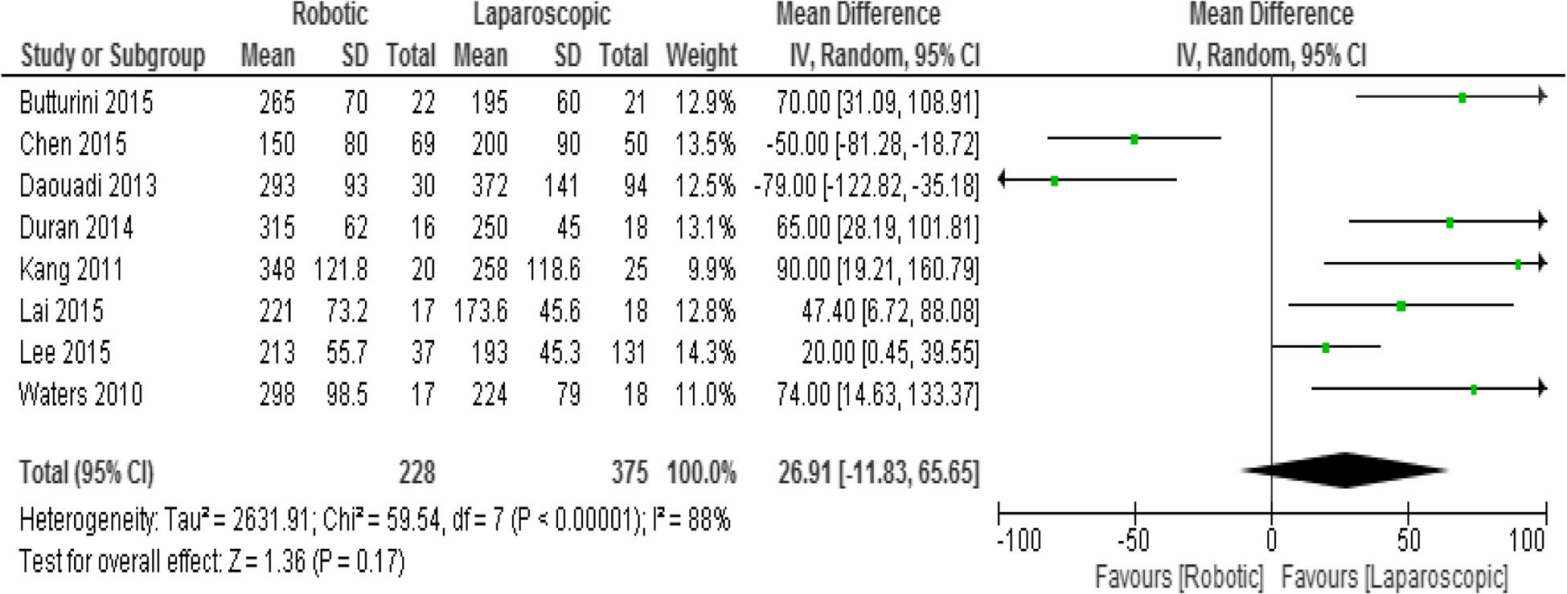
Forest plot displaying the results of the meta-analysis regarding operative time

Eight studies reported the operative time which was respectively 262.8 min in the RDP group and 233.2 in the LDP group. The difference between the two groups was not statistic significant (mean difference = 26.91 95% CI -11.8 + 65.6; *p* = 0. 17), Fig. 7.

## Discussion

This meta-analysis demonstrates the safety and feasibility of the robotic approach to distal pancreatectomy. Specifically, the results of our study reveal that RDP does not increase the rate of post-operative complications, is associated to higher rate of spleen preservation, reduces hospital stay and decreases conversion rate.

Many studies of minimally invasive distal pancreatectomy have been published in the literature, highlighting the increasing surgical community interest in this new technique [33, 34].

Several studies have compared open versus laparoscopic distal pancreatectomy, demonstrating the superiority of the latter in terms of less blood loss, faster recovery and less hospital stay [10, 35–37].

Robotic surgery is the latest development of mini-invasive surgery of the pancreas. This technology maintains the advantages of laparoscopic technique in terms of smaller surgical scars and faster functional recovery, but adds the specific advantages of robotic surgery; in fact, thanks to the stability of articulated instruments and magnification of the 3D high definition view, the robot allows more complex surgical operations to be performed. This added value of RDP could increase the chance to increase the rate of spleen preservation [38, 39].

This meta-analysis shows that the RDP increases the rate of splenic preservation; in fact, 7 studies indicated a better spleen preservation rate through robotic surgery. The preservation of the spleen has been shown to be important in preventing postoperative complications and particularly the overwhelming post-splenectomy infection syndrome. The preservation of the spleen, however, depends not only on technical factors but primarily on the indication for pancreatectomy. In fact, malignant tumors are not an indication for the conservation of the spleen, which is instead generally taken into account for benign or neuroendocrine tumors [40]. Two spleen-preservation surgical techniques have been described: the Kimura [41] and the Warshaw method [42]. In the Kimura’s technique the artery and splenic vein are skeletonized and preserved in order to maintain the vascular flow to the spleen. The Warshaw method consists in the section of splenic vessels while preserving short gastric vessels and left gastroepiplonic artery, which provide adequate vascular flow to the spleen. This second technique seems to increase the risk of spleen infarction. The studies considered in our review do not provide the details of the surgical technique used to preserve the spleen and therefore a comparison between the two methods has not been performed [43].

With regard to overall postoperative complications, these were similar between two groups. The most frequently reported complication was intra-abdominal fluid collection. However, severe complications, defined as Dindo-Clavien ≥ 3, were similar in the two groups, as was the reported mortality.

The pancreatic fistula is still the Achilles heel of pancreatic surgery [44–46]. This complication remains a very serious problem because it increases morbidity and lengthens hospital stay. Regardless of the technique used to cut and close the pancreatic stump, the incidence of postoperative pancreatic fistula varies from 0 to 47% [47]. A recent meta-analysis compared different methods of treating pancreatic parenchyma after distal pancreatectomy, but none of the techniques used was superior to the others in reducing the incidence of pancreatic fistula [48]. In this meta-analysis, nine studies compared the rate of pancreatic fistula between RDP and LDP failing to show any significant differences. In particular, the rate of severe pancreatic fistulas grade B / C was not statistically different between the two groups.

Blood transfusion during surgery for malignant disease is associated with an increased risk of long-term relapse [49]. In the meta-analysis we did not observe statistically significant differences in the rate of blood transfusions between the two groups.

In relation to oncological parameters, we did not observe significant differences in the considered studies. It was interesting to note that the surgical margins were negative (R0) with a near 100% rate in the two groups and good lymphadenectomy was performed in both groups. However, no indication was provided by the authors regarding chemo-radiotherapy treatments with adjuvant or neoadjuvant purposes. No specific data were also available regarding disease free survival and tumor recurrence. Therefore it is not possible to draw final conclusion on the oncological adequacy of the robotic approach in this type of surgery.

Since minimally invasive surgery is typically associated with a faster recovery, the length of hospital stay is a very important index in the evaluation of this type of surgical approach [38, 50].

A shorter hospital stay was observed in the RDP group in our study. This result could be an argument in favor of robotic surgery in reducing the overall impact of the cost, which is still considered very high by several authors. Each robotic procedure generally costs from 1000 to 3000 dollars more than a laparoscopic procedure. Our meta-analysis shows that the robotic procedure is more expensive than the laparoscopic one. However, in calculating the cost of the operation Waters et al. [26] took into account the associated cost of the hospital stay. In this case, robotic surgery showed a greater economic advantage over laparoscopic surgery with an estimated cost of 10,588 and 12,986 dollars respectively for the RDP and the LDP group. It should be noted also that prices often vary considerably among the different surgical centers, even in the same country, so the this comparison may be misleading and at risk of bias.

Our systematic review summarizes most of the available evidence in this context. However, it has some limitations. Although most of the included studies showed a high methodological quality according to the Newcastle-Ottawa scale, the studies were retrospective and not randomized. The absence of randomization and the retrospective nature involves some structural bias that could lead to inaccurate or incorrect conclusions. Further prospective randomized studies are therefore needed to understand which of the two methods is superior to the other in terms of cancer, complications and long-term results.

## Conclusions

This meta-analysis suggests that the RDP procedure is safe and comparable in terms of surgical results to LDP. However even if the RDP has a higher cost than the LDP, it increases the rate of preservation of the spleen, reduces the risk of conversion to open surgery and is associated to shorter length of hospital stay. Nevertheless, due to the high risk of bias of these retrospective studies, the benefits of RDP proven in our meta-analysis should be confirmed through further RCTs.

## Abbreviations

C.I.: Confidence interval
DP: Distal pancreatectomy
LDP: Laparoscopic distal pancreatectomy
MD: Mean difference
OR: Odds ratio
PF: Pancreatic fistula
RDP: Robotic distal pancreatectomy
